# Letter to the Editor: Use of PDRN and Nd:YAG Q‐Switched 1064 nm Laser in the Management of Post‐Inflammatory Hyperpigmentation Following Laser Hair Removal Complication

**DOI:** 10.1111/jocd.70573

**Published:** 2025-11-27

**Authors:** Rafael Rodrigo Crisanto de Oliveira

**Affiliations:** ^1^ Centro De Ciências Da Saúde, Universidade Federal Da Paraíba João Pessoa Brazil

Dear Editor,

Laser hair removal has become one of the most widely used modalities in esthetic dermatology, with recognized efficacy in reducing unwanted hair. Different technologies and wavelengths allow treatment to be tailored according to skin phototype and body area [1]. However, complications may occur, particularly in cases of exaggerated inflammatory response, evolving with pain, burns, and subsequent post‐inflammatory hyperpigmentation (PIH), a condition that remains challenging to manage.

Polydeoxyribonucleotide (PDRN) is a DNA derivative with regenerative, anti‐inflammatory, and depigmenting properties. Obtained through purification to minimize immunological risks, it acts via activation of A2a receptors, providing nucleotides for DNA synthesis and promoting tissue repair. Experimental and clinical studies have already demonstrated its efficacy in accelerating wound healing, improving the quality of skin regeneration, and exerting brightening and anti‐inflammatory effects [2, 3].

The Nd:YAG Q‐Switched 1064 nm laser emits ultrashort, high‐energy pulses capable of fragmenting melanin deposits in different layers of the skin, facilitating their clearance by the lymphatic system. Through this mechanism, it has been widely used in the treatment of several pigmentary disorders, including melasma, Becker's nevus, and argyria [4]. The association of this technology with PDRN has been previously described in a report of PIH induced by 20% trichloroacetic acid, with a satisfactory cosmetic outcome, supporting its application in similar scenarios [5].

In the present case, on 05/16/2025, a young and previously healthy patient underwent laser hair removal using a multi‐wavelength platform equipped with Ruby (694 nm), Alexandrite (755 nm), Diode (808 nm), and Nd:YAG (1064 nm) wavelengths (medium mode, fluence 18 J/cm2). This specific combination and mode are a pre‐programmed setting suggested by a popular multi‐wavelength machine in Brazil, which associates all four wavelengths in its standard configurations. The hair removal was done only on the anterior torso.

Although no immediate pain was reported, a few hours after the procedure the patient developed intense erythema, diffuse perifollicular edema, severe pain, and a burning sensation on the chest and abdomen. Clinical examination revealed widespread erythema, with a risk of blister formation and residual hyperpigmentation. Initial management included intravenous corticosteroids, systemic analgesics, and topical barrier protection.

On reassessment (05/20/2025), the condition had evolved into PIH, characterized by multiple diffuse brownish macules, some with central clearing. On 05/24/2025, topical PDRN was introduced, with progressive improvement in wound healing. Figure 1 shows the post‐complication sequence, with a symmetrical punctate burn pattern that rapidly evolved into hyperpigmentation, predominantly in parasternal and periareolar regions.

**FIGURE 1:**
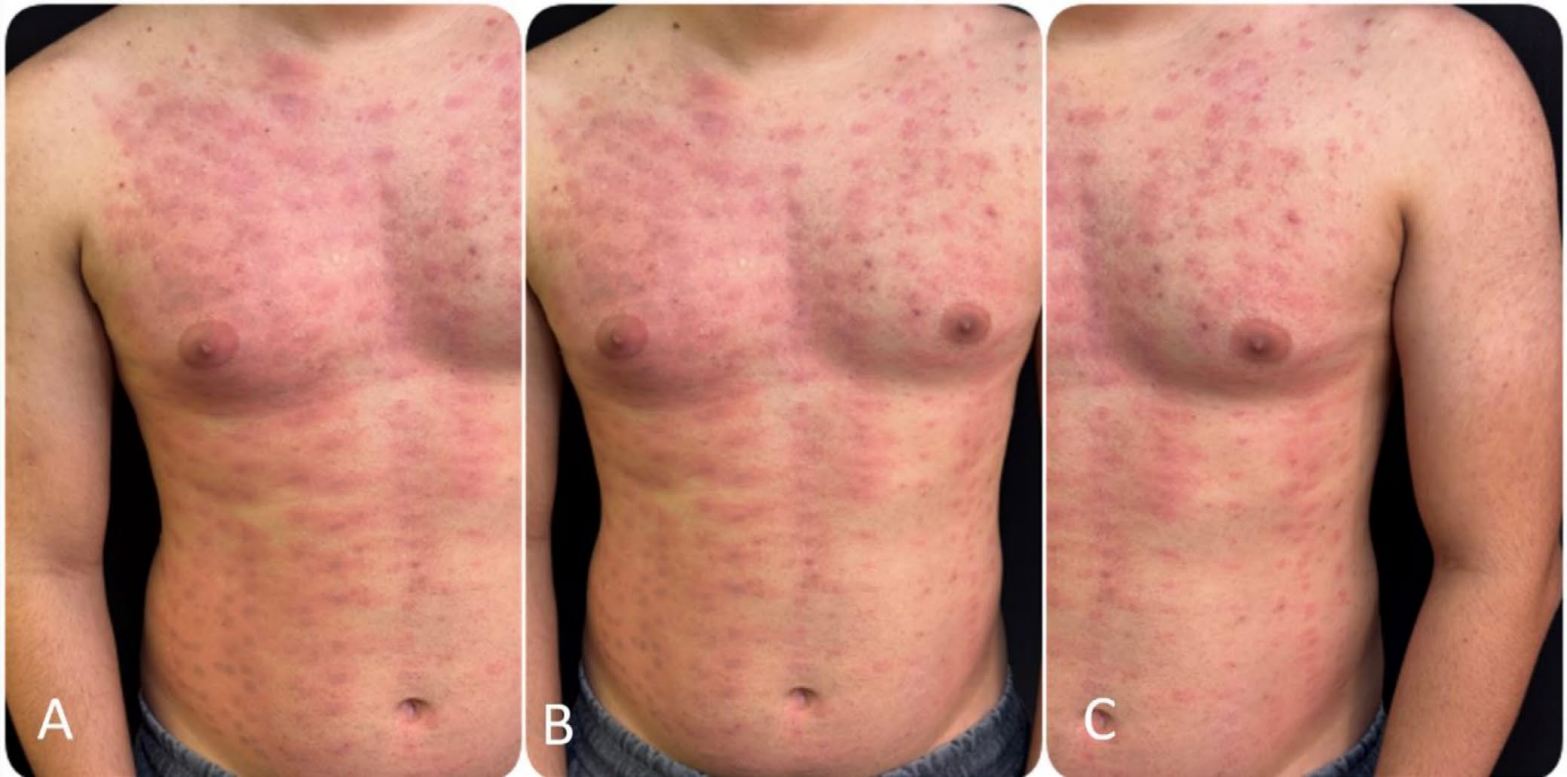
Clinical documentation of the complication after laser hair removal. (A–C) Images taken on 05/16/2025 showing erythema, perifollicular edema, and initial burn pattern on the chest and abdomen.

On 08/19/2025, residual hyperpigmented plaques persisted, prompting the performance of a black peel session (using a carbon‐based lotion) using Nd:YAG Q‐Switched 1064 nm (6 mm spot, 2 J/cm^2^), followed by reapplication of topical PDRN right after the procedure. For home care, a moisturizing vehicle containing dexpanthenol 5%, niacinamide 4%, and shea butter 1% was prescribed. By 09/23/2025, a marked lightening of the macules was observed, with satisfactory cosmetic results. Figure 2 illustrates the clinical course: initial regression of erythema (05/20–05/24), partial lightening of the lesions (08/19), and overall satisfactory esthetic outcome (09/23).

**FIGURE 2:**
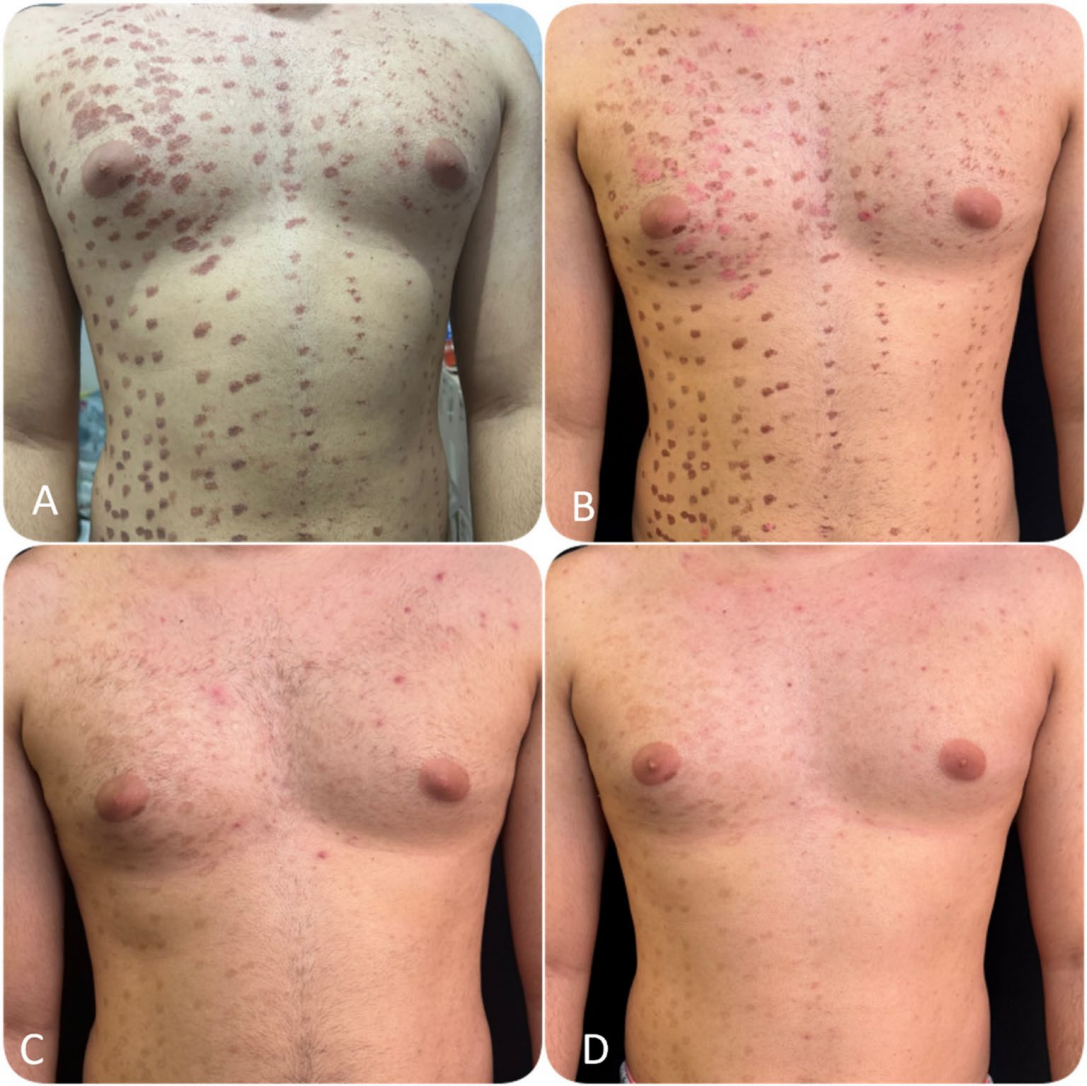
Sequential clinical evolution following therapeutic interventions. (A) 05/20/2025: Development of diffuse brownish macules consistent with PIH. (B) 05/24/2025: Progressive healing and initial lightening after topical PDRN. (C) 08/19/2025: Persistence of hyperpigmented plaques before black peel session with Nd:YAG Q‐Switched 1064 nm. (D) 09/23/2025: Marked improvement with significant lightening of lesions and satisfactory cosmetic outcome.

Figure 1 illustrates the acute presentation of the complication, characterized by diffuse erythema, perifollicular edema, and subsequent development of post‐inflammatory hyperpigmentation in a punctate and symmetrical pattern over the chest and abdomen. This highlights the severity of the inflammatory response and the high risk of persistent pigmentary sequelae. Conversely, Figure 2 documents the progressive improvement after combined therapy with topical PDRN and Nd:YAG Q‐Switched 1064 nm laser. Over sequential follow‐up, there is a visible reduction in pigmentation intensity, greater homogeneity of skin tone, and improved cosmetic appearance. The comparative analysis underscores the therapeutic potential of this multimodal approach in managing PIH after laser hair removal.

Although it is not possible to determine the isolated contribution of each intervention, this case highlights both the complexity of laser hair removal complications and the therapeutic potential of combining PDRN's regenerative and anti‐inflammatory effects with the Nd:YAG Q‐Switched 1064 nm's targeted melanosome fragmentation. Sequential photographic analysis demonstrates progressive pigment reduction, improved skin uniformity, and increased patient comfort.

Despite these encouraging findings, the literature still lacks robust evidence regarding the combined use of these approaches. Prospective, controlled, and larger‐cohort studies are warranted to validate the efficacy and safety of this association and to establish standardized protocols to better guide clinicians in similar situations.

## Ethics Statement

The author confirms that the ethical policies of the journal, as noted on the journal's author guidelines page, have been adhered to. No ethical approval was required as this is a review article with no original research data.

## Conflicts of Interest

The author declares no conflicts of interest.

## Data Availability

The data that support the findings of this study are available on request from the corresponding author. The data are not publicly available due to privacy or ethical restrictions.
